# Effect of Feeding 0.8% Dried Powdered *Chlorella vulgaris* Biomass on Growth Performance, Immune Response, and Intestinal Morphology during Grower Phase in Broiler Chickens

**DOI:** 10.3390/ani12091114

**Published:** 2022-04-26

**Authors:** Simon Roques, Sietse-Jan Koopmans, Annemarie Mens, Jan van Harn, Marinus van Krimpen, Soumya Kanti Kar

**Affiliations:** Wageningen Livestock Research, Wageningen University and Research, 6708WD Wageningen, The Netherlands; simon.roques@wur.nl (S.R.); sietsejan.koopmans@wur.nl (S.-J.K.); annemarie.mens@wur.nl (A.M.); jan.vanharn@wur.nl (J.v.H.)

**Keywords:** broiler chicken, *Chlorella vulgaris*, haptoglobin, immune response, interleukin-13, histomorphology

## Abstract

**Simple Summary:**

*Chlorella vulgaris* (CV) is a microalgae of great interest for broiler production, due to its nutritional and functional properties. Currently, expensive bioprocessing steps are used to acquire the functional components that could achieve the desired biofunctionality of CV, in particular to maintain productivity and support health in broilers. This makes CV an expensive feed ingredient that can only be used in low inclusion levels, otherwise the cost of the feed is too high. However, it is not clear whether low CV inclusion levels have positive effects on broiler production and immune response. This study investigated whether the addition of CV biomass, at a low inclusion level of 0.8%, to broiler diets maintained the functional properties of microalgae and improved broiler productive performance. We found that the addition of 0.8% CV biomass affects the immune response, and has positive effects on the overall productive performance of broilers.

**Abstract:**

An experiment was performed to study the effects of a low inclusion level of *Chlorella vulgaris* (CV) biomass in broiler diets on performance, immune response related to inflammatory status, and the intestinal histomorphology. The study was performed with 120 Ross 308 male broiler chickens from 0–35 days of age. The broilers were housed in 12 floor pens (1.5 m^2^) bedded with wood shavings. The broilers received a three phase diet program, either with 0.8% CV biomass (CV) or without CV (CON). Each diet program was replicated in six pens. The final body weight increased (*p* = 0.053), and the feed conversion ratio (FCR), corrected for body weight, was reduced (*p* = 0.02) in birds fed CV compared to birds fed CON. In addition, decreased haptoglobins (*p* = 0.02) and interleukin-13 (*p* < 0.01) responses were observed during the grower phase of birds fed CV compared to the birds fed CON. A strong correlation (r = 0.82, *p* < 0.01) was observed between haptoglobin response and FCR. Histomorphology parameters of the jejunum were not different between the groups. It was concluded that the inclusion of 0.8% CV biomass in broiler diets is effective in influencing immune responses related to inflammatory status and promoting broiler growth.

## 1. Introduction

*Chlorella vulgaris* (CV), a species of unicellular freshwater microalgae, is of interest for broiler production, mainly due to its nutritional properties [1,2,3]. The quantitative and qualitative macro- and micronutrient composition of CV makes it a relevant ingredient for broiler diets [4,5]. *Chlorella vulgaris* generally contains more than 50% protein [6] and can be used in broiler diets as a partial or complete substitute for protein sources, such as fish meal (up to 5%) and soybean meal (up to 10%), without negative effects on weight gain or the feed conversion ratio (FCR) [2,7]. Based on these characteristics, CV clearly qualifies as a feasible ingredient in broiler diets [8,9], but high levels of CV are not economically viable [10], and the indigestible cell wall might prevent access to valuable nutrients [9]. Further, CV is attractive to feed producers due to its environmental impact. It provides an environmentally friendly alternative to traditional crop-based feed ingredients, as it does not compete for the same land area used for food production, and also serves as a natural sink for carbon dioxide [11].

In addition to providing ‘strict-nutritional’ components, CV also provides ‘non-strict-nutritional’ or functional properties [12]. Biochemical constituents such as carotenoids, β-glucan, phenolic compounds, phytosterols, and peptides in CV are reported to confer immune-modulatory properties, antioxidant activity, and support gut tissue regeneration, along with improved productive performance, in livestock [2,5,13,14,15,16,17]. However, expensive bioprocessing steps are required to acquire these functional components of CV. Animal nutritionists aim to use low doses of CV products, such as polysaccharides or protein extract from bioprocessing steps of the biomass, primarily to keep the cost of a CV-based diet comparable to current feed formulations for broiler chickens. A study shows that the inclusion of 1% CV product in a broilers diet could replace antibiotics in the feed, along with having an impact on the gut microbiota composition that promotes the health and productivity of broiler chickens [18]. However, it is desirable to include CV biomass in broiler diets as an alternative to expensive CV products. Unlike CV products, no complex and expensive bioprocessing steps are required to obtain CV biomass. Researchers show that adding fermented CV [19], or dried or fresh CV powder [4,18], at a dosage of 1–2% into broiler diets improves growth performance, modulates immune response, and affects the intestinal tissue morphology in broilers. These studies [18,19] confirm the use of CV biomass at low dosages in broiler diets, which confers the functional properties attributed to CV products in other studies [2,5,13,14,15,16,17,20]. It is economically valuable for farmers to use CV biomass at even lower dosages, if the functional properties are conserved. Therefore, the aim of this study was to investigate whether the inclusion of 0.8% CV dried, powdered biomass alters productive performance, immune responses related to inflammatory status, and the intestinal histomorphology in broiler chickens.

## 2. Materials and Methods

### 2.1. Animals, Design of Experiment and Housing

The study was approved by the animal welfare body at Wageningen University & Research (WUR; experiment number 2016.D-0065.013) and the Dutch Central Committee of Animal Experiments (The Hague, The Netherlands; protocol number AVD401002015196). All procedures were carried out according to the guidelines of the European Council Directive 2010/63. The study was performed over 35 days with 120, one-day old Ross 308 male broiler chicks obtained from a commercial hatchery (Probroed & Sloot hatchery, Meppel, The Netherlands). The broilers were housed in 12 floor pens of 1.5 m^2^ bedded with white wood shavings. These pens were located in two rooms of the mechanically ventilated animal facility of WUR, Lelystad, The Netherlands.

The broiler chickens were randomly divided into two dietary treatment groups, i.e., 0.8% CV biomass (CV) or without CV biomass (CON). Each dietary treatment was replicated six times, with a floor pen as an experimental unit, resulting in 6 pens of 10 birds per dietary treatment evenly allocated per room (3 CV and 3 CON pens per room).

The ambient temperature upon arrival of the one-day old chicks to the experimental facility was 34 °C, which was gradually reduced by 1 °C until day 7; thereafter, the ambient temperature was gradually reduced to 21 °C by day 28, and this temperature was maintained until the end of the experiment. Artificial light, with an intensity of 20 lux, was provided throughout the experimental period, with continuous light (24 L:0 D) for the first two days. From day 3 until the end of the experiment, a day/night schedule of 18 h light and 6 h dark (18 L:6 D) was maintained. The birds were vaccinated against infectious bronchitis (Nobilis® IB MA5, Intervet, Boxmeer, The Netherlands) on arrival, and against Newcastle disease (Nobilis® ND clone30, spray vaccination coarse drop, Intervet, Boxmeer, The Netherlands) on day 14.

### 2.2. Chlorella Vulgaris Biomass

The CV was cultured and biomass was provided by Phycom (Veenendaal, The Netherlands). The CV culture was axenic, i.e., not contaminated with other microorganisms, and was grown in a closed environment. After harvesting, the water was separated, creating an algae paste. The algae paste was then dried by a drum dryer. The drying temperature and speed were optimized to maintain the nutritional profile of the microalgae. The dried microalgae were then ground to a fine powder and immediately packed in a light-proof and airtight package. The nutritional composition of the CV biomass is presented in Supplementary Table S1.

### 2.3. Diets and Feeding

All diets were formulated to meet or exceed the Centraal Veevoeder Bureau recommendations for broiler chickens [21]. The birds were fed a 3-phase customized isocaloric (based on metabolizable energy) diet, with a similar digestible essential amino acid profile per feeding phase: starter (day 0–14), grower (day 14–28), and finisher phase (day 28–35). In all phase diets, the birds received either 0.8% CV biomass (CV) included in the diet, or no CV (CON). The ingredients and chemical compositions of the experimental diets are presented in Supplementary Tables S2 and S3. During the whole experimental period, the birds had ad libitum access to the experimental feed provided in feeding troughs, and water via nipple drinkers (three nipples/pen).

### 2.4. Broiler Performance

The body weight (BW) of the birds was measured per pen on days 14, 28, and 35. Total feed intake per pen was determined as provided feed minus remaining feed for each feeding phase, and for the whole experimental period.

Daily body weight gain (dBWG) per bird was calculated as follows:

dBWG = [(BW end period/*n*) − (BW start period/*n*)]/number of days (1)

where *n* is the number of birds at the start or end of the period.

The feed conversion ratio (FCR) for each phase, and for the whole experimental period, was calculated as follows:

FCR = total feed intake period/(BW end period − BW start period)(2)

Daily feed intake (dFI) per bird for each phase, and for the whole experimental period, was calculated as follows:

dFI = (FCR × BWG)/number of days(3)

Over the whole experimental period, the FCR, corrected at a standard weight of 2250 g (FCR_corrected_), was calculated. The FCR_corrected_ is a metric that allows the comparison of feed conversion ratio for a same weight [22,23,24]. It is useful to normalize FCR when differences of body weight occur, as the FCR is sensible to body weight differences. The correction used was 0.02 FCR points per 100g weight difference. The FCR_corrected_ was calculated as follows:

FCR_corrected_ = FCR_0–35_ − ((BW_35_ − 2250)/50 × 100)) (4)

where FCR_0-35_ represents the FCR over the whole experimental period and BW_35_ represents the final body weight at the end of the 35 days. All performance indicators were averaged by dietary treatment.

### 2.5. Immune Responses

Two birds per pen (*n* = 12 per diet) were color marked based on their BW. These corresponded with the pen’s average BW on the first day of sampling (day 14). On days 14 and 21, blood samples were collected from the wing veins of the marked birds and serum was extracted. Blood samples were collected in sterile Vacuette tubes containing Z serum separator clot activator (Greiner Bio-One B.V., Alphen aan den Rijn, The Netherlands), and the tubes were gently inverted and allowed to clot for at least 30 min. All tubes were centrifuged at 2200× *g* for 15 min at 20 °C and serum was extracted. The serum were stored at −20 °C for further analysis of the immune markers haptoglobin (Hap), interleukin-13 (IL-13), and interferon-gamma (IFN-γ). These immune markers were measured in the serum samples using the Haptoglobin ELISA kit (CHK011) (ABclonal via Diversified Biotech B.V., Ulvenhout, The Netherlands), Chicken IFN-γ ELISA kit (ABIN1563358), and Chicken IL-13 ELISA kit (ABIN1563404) (both from Elabscience via Antibodies-online, Aachen, Germany). Manufacturers protocols were followed to measure the concentrations levels. Briefly, Hap concentrations were measured using colorimetry with electromagnetic radiation absorbance at 450 nm, based on the competitive ELISA of an Hap-Horseradish peroxidase conjugate. For IFN-γ and IL-13, concentrations were measured using a sandwich-ELISA principle, with biotinylated detection antibody specific for the cytokines and Avidin-Horseradish peroxidase conjugate. The measured immune responses were calculated at individual levels, using area under the curve (AUC) between day 14 and 21, according to the method of the trapezoidal rule [25].

### 2.6. Intestinal Histomorphology

On day 14, two birds were randomly selected per pen (*n* = 12 per diet) and euthanized by electrocution. Immediately after euthanasia, the gut was dissected. Briefly, about 5–10 cm anterior to Meckel’s diverticulum was marked as the sampling site to obtain jejunal tissue. To preserve the micro-architecture of the villi and crypts, 1–2 mL of 4% buffered formaldehyde solution was injected right next to the sampling site, and 2 cm long segments were collected. The collected samples were then fixed and stored in vials containing 4% formaldehyde solution, and the vials were kept at room temperature until further analysis. Intestinal histomorphology was assessed by Gezondheidsdienst voor Dieren B.V. (Royal GD, Deventer, The Netherlands). Briefly, formalin-fixed tissues were dehydrated in a graded series of ethanol, cleared in xylene, and embedded in paraffin wax. For each segment, a 2 μm cross-section was made using a microtome, and stained according to the hematoxylin–eosin method. Images were viewed using a light microscope (Olympus BX41) connected to an Olympus DP26 camera, in order to measure morphometric parameters using Olympus cellSens Dimension software version 1.12. The villus length and crypt depth were measured from 10 intact and well defined villi and adjacent crypt for each tissue section. To determine the villus length, the distance between the tip of the villus to the villus–crypt junction was measured, and for crypt depth, the distance from the villus–crypt junction to the lower limit of the crypt. The average values of these measurements per tissue section were used in the statistical analysis. Furthermore, from the same slides, the number of goblets cells were counted and the total area of all these goblets cells, expressed in µm^2^, was measured. For each slide the total goblet cell area was expressed as a percentage of the villus area, and the average goblet cell size was determined per villus.

### 2.7. Statistical Analysis

Diet effect was analyzed using the Student’s *t*-test on all the measures and calculated metrics of this trial. Normality was assessed using the Shapiro–Wilk test, and the homoscedasticity by visual inspection of the residual vs. fitted plot. For growth performance, the pen was the experimental unit (*n* = 6 per diet). For immune parameters and histomorphology, individual birds were the experimental unit (*n* = 12 per diet). Interleukin-13 and IL-13 AUC were log transformed, as distributions were not normal, as was haptoglobin on day 14. To examine any relationship between immune parameters and feed efficiency, Pearson correlation was performed with R software [26] separately between the average value of Hap or IL-13 AUCs per pen, and the FCR per pen for the whole experimental period (day 0 to 35). A *p*-value < 0.05 was defined as a significant threshold, and a *p*-value < 0.1 was considered as trend threshold (indicating tendency).

## 3. Results

The effects of feeding a 0.8% dried powdered CV-based diet on growth performance, immune response, and the intestinal morphology of broiler chickens in the grower phase are summarized in Table 1.

### 3.1. Production Performances

The effects of CV inclusion in diet on BW, dBWG, FCR, dFI, and FCR_corrected,_ over the whole experimental period, are presented in Table 1. The final BW increases in the CV group by 3.9%, compared to the CON group (*p* = 0.053). A similar trend is also reflected in the dBWG over the whole experimental period (+3.8% in the CV group compared to CON group, *p* = 0.053). The average dFI and FCR over the whole experimental period do not differ significantly between the dietary treatments (*p* = 0.25 and *p* = 0.23, respectively). However, the FCR_corrected_ is significantly reduced in the CV group compared to the CON group (1.39 vs. 1.42; *p* = 0.02). There were no significant effects on BW, dBWG, FCR and dFI between the CV and CON groups during each individual phase.

### 3.2. Immune Responses

**Figure 1:**
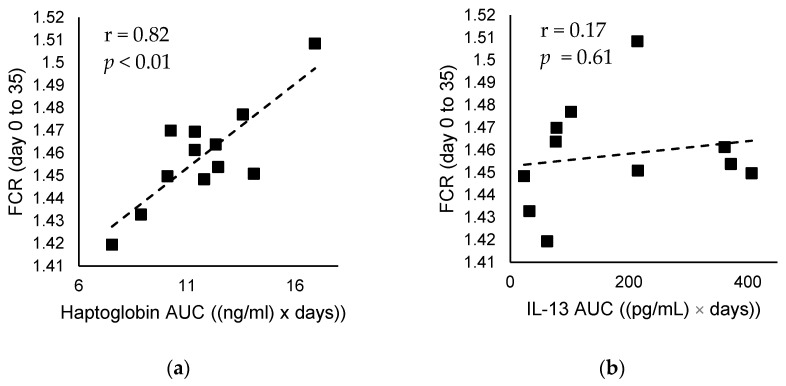
Correlation between measures of immune response and feed conversion ratio (FCR) over the whole experimental period (day 0 to 35). (**a**) Haptoglobin AUC against feed conversion over the whole experimental period, (**b**) Interleukin-13 AUC against feed conversion over the whole experimental period. r, Pearson correlation coefficient; *p*, *p*-value.

Haptoglobin and IL-13 AUC between day 14 and 21 are presented in Table 1. The calculated AUC for haptoglobin is significantly reduced by 20.90% (*p* = 0.02), while the calculated AUC for IL-13 is significantly reduced by 39.10% in the CV group compared to CON group (*p* < 0.01). Haptoglobin and IL-13 concentrations on days 14 and 21 are presented in Table 1. The haptoglobin concentration on day 14 is reduced by 22% (*p* = 0.09), and on day 21 is reduced by 14% in the CV group compared to CON group (*p* = 0.06). Interleukin-13 on day 14 is significantly reduced by 86% (*p* < 0.01), and on day 21 is not significantly changed in the CV group compared to CON group (*p* = 0.41). Furthermore, the correlations calculated indicate a significant strong correlation between haptoglobin AUC and FCR (Figure 1a) (r = 0.82, *p* < 0.01), but not between IL-13 AUC and FCR (r = 0.17, *p* = 0.61) (Figure 1b).

### 3.3. Histomorphology

The histomorphology characteristics, measured on day 14, of jejunum in birds fed CON or CV diets are presented in Table 1. Measured values for villi length (*p* = 0.32), crypt depth (*p* = 0.27), villi/crypt (V:C ratio) (*p* = 0.15), villi size (*p* = 0.21), goblet cell surface area (*p* = 0.85), goblet cells per villi (*p* = 0.54), and goblet cell size (*p* = 0.62) do not differ between CON and CV groups.

## 4. Discussion

The aim of this study was to investigate the effects of CV dried powdered biomass, at a dosage of 0.8%, on production performance, immune response related to inflammatory status, and the intestinal histomorphology of broiler chickens.

In our study, pens of 1.5 m^2^ were used, housing 10 broilers each. Theis number of birds is not comparable to practice, where the number of birds and stocking density are much higher. The stocking density in this study (approx. 13.9 kg/m^2^) is much lower compared to broiler farm regulations (39–42 kg/m^2^). However, this study aimed to assess the physiological and biological effects of CV, not test its applicability in practical conditions.

The inclusion of CV biomass in broiler diets during the 35 days of the trial tended to improve the final BW of the birds. This result is in agreement with most previous studies, in which inclusion of less than 1% of biomass from CV improved the BW of the birds [4,18,27,28], with the exception of one study [29]. Thus, our results confirm positive effects of CV biomass at a low dose on broiler BW. However, in the present study, FCR was not affected by CV diet. This result is in agreement with [18,27,28] but not with [4,29], where FCR was reduced. The discrepant results could be due to several reasons, such as differences in the microalgae strain, inclusion level, and variations in experimental design in the conducted trials. Researchers often correct the FCR for weight differences and calculate the corrected FCR, which provides a reliable comparison of dietary treatments at the same body weight [22,23,24]. In our case, the corrected FCR was reduced in the CV group, indicating that the trend of higher body weight gain is due to better feed efficiency. Thus, our study confirms that the CV-based diet has a positive effect on productive performance, even when the CV biomass is supplied at a low level. Considering the low quantity of nutrients supplied by the CV biomass in the broiler diets, it can be assumed that these are the functional properties of CV biomass that are responsible for the better production performance [5,14]. To determine whether CV biomass has functional effects at low doses, immune response and the intestinal morphology were studied during the grower phase in order to gain insight into the mechanisms involved in growth.

We reported the effect of CV biomass at low levels in broiler chickens on the acute phase protein Hap, and pro-inflammatory cytokines IFN-γ and IL -13, based on the evidence of immunomodulatory effects of CV observed in broiler chickens [18], mice [30], and in in vitro studies [31]. It is likely that the beneficial effects of diet on performance in later life in broiler chickens have a carry-over effect from the impact of the dietary treatment on (gut) health, in particular on the immune system during the (critical) early post-natal rearing period of broilers chickens [32]. Due to the short lifespan of broiler production practices, it is easier and faster to study the effects of CV on the whole productive cycle of early life events. Taking this advantage, we focused on evaluating the impact of a CV-based diet on broiler chickens on the immune response, by measuring concentration levels of serum inflammatory markers on day 14 and day 21 of this feeding trial, as compared to FCR calculated from 0-35 d. The undetectable IFN-γ level in both groups confirms the absence of a strong inflammatory response mounted by the birds throughout the feeding trial, as IFN-γ typically responds to acute and short-term high-potency stimuli, such as pathogens or exposure to potent immunogens [33,34]. To the best of our knowledge, this is the first report in which broiler chickens fed CV biomass show a suppressed response of Hap and IL-13. However, the suppressed production of IL-13 is in agreement with other studies conducted on mice [35] and humans [36] treated with CV products. The reason for of the reduction of Hap and Il-13 response in serum could be a direct effect of various bioactive components in microalgae on immune cells, such as xantophylls [37,38], phytosterols [38,39], PUFA [40], peptides [41], and polysaccharides [20].

In chickens, an activated immune system requires a supply of nutrients from the diet, thus, limiting the supply for productivity, which negatively affects BWG and FCR [42,43]. For example, stressors such as an unbalanced diet, dysbiosis, and disease divert nutrients from the diet from growth to immunity, to negate the stressor. During stress, nutrients such as amino acids, normally used for growth and productivity in broiler birds, are directed to immune cells to promote the production of immune proteins that induce cytokines and acute phase proteins in the liver, including Hap [44,45]. The strong correlation between FCR and haptoglobin in broiler chickens suggests that birds fed CV biomass elicit a favorable immune response that allows metabolism to be adjusted to an efficient mode of nutrient utilization, in which nutrients and energy expenditure are directed toward growth and productivity. However, no correlation was found between FCR and IL-13 response, suggesting that IL-13 does not have a major impact on broiler performance. These results suggest that lower inclusion rates of CV biomass influence immunity in a manner that supports broiler productive performance. It is important to understand such nuances of nutrition and immunity, in order to optimize bird health and productivity, as this can go a long way in meeting consumer demands for more natural production, and improved animal welfare [45].

In this study, we also measured structural changes in intestinal morphology by histomorphology of jejunum on day 14. We hypothesize that this measure may partially reflect intestinal functionality, which could be altered by CV inclusion, and, similarly to immune response, we consider the beginning of the grower phase a critical phase for the lifespan of broilers. Jejunum is considered the main site of nutrient uptake from the diet, with villus height providing an estimate of the surface area available for nutrient uptake, and crypt depth providing an indication of the rate of villus renewal. In addition, goblet cells are secretory cells that produce mucin to form a protective mucus layer. In this study, the histomorphology of broilers fed CV biomass was not altered compared to CON fed broilers. Other researchers report a significant increase in villus height and/or crypt depth in broilers fed CV [15,46], but the diets contained CV products, and not CV biomass. Moreover, in the reported experiments, intestinal morphology was performed on tissue samples collected at the later age of broilers fed with CV, i.e., 35 days [46] and 42 days [15], and in the case of Kang et al. (2017) [46], morphology analysis was performed in the ileal section, and not in the jejunum as in our case. Thus, to conclude that CV biomass is less effective on intestinal morphology than CV product is too speculative. However, the similar morphology of the jejunum in broiler chickens fed the experimental diets suggests that the improved growth performance in the present study is not due to better nutrient absorption. The latter supports the hypothesis that CV biomass improves broiler performance by alleviating the immune system, rather than by improving nutrient uptake.

## 5. Conclusions

In conclusion, the inclusion of 0.8% of CV biomass in broiler diets has a significant effect on bird immunity, and improves body weight by day 35, with significantly improved FCR_corrected_. These benefits of CV biomass suggest that immune function related to inflammatory status is maintained at low inclusion levels, and that could eventually improve the competitiveness and economic prospects of CV as a sustainable feed additive for broiler diets.

## Figures and Tables

**Table 1:** Dietary effect of feeding either a control (CON) or *Chlorella vulgaris* (CV)-based diet ^1^ on growth performance, immune response, and intestinal morphology of broiler chickens.

Measured Parameters		CON	CV	*p*-Value ^2^
	Performance parameters ^3^ of broilers during day 0–14			
Growth performance	BW start (g) on day 0	46.9 ± 1.0	47.1 ± 0.6	0.70
	BW end (g) on day 14	481.4 ± 34.6	482.9 ± 39.2	0.95
	dBWG (g/d)	31.0 ± 2.5	31.1 ± 2.8	0.96
	FCR (g/g)	1.21 ± 0.01	1.21 ± 0.02	0.76
	dFI (g/d)	41.6 ± 3.2	41.8 ± 3.8	0.91
	Performance parameters ^3^ of broilers during day 14–28			
	BW start (g) on day 14	481.4 ± 34.6	482.9 ± 39.2	0.95
	BW end (g) on day 28	1738.6 ± 85.4	1787.3 ± 73.9	0.36
	dBWG (g/d)	90.1 ± 5.1	93.3 ± 3.3	0.27
	FCR (g/g)	1.38 ± 0.02	1.36 ± 0.02	0.30
	dFI (g/d)	123.9 ± 6.2	127.2 ± 4.4	0.35
	Performance parameters ^3^ of broilers during day 28–35			
	BW start (g) on day 28	1738.6 ± 85.4	1787.3 ± 73.9	0.36
	BW end (g) on day 35	2509.5 ± 75.9	2606.3 ± 63.2	0.05
	dBWG (g/d)	110.1 ± 7.9	117.0 ± 6.2	0.16
	FCR (g/g)	1.69 ± 0.10	1.65 ± 0.08	0.52
	dFI (g/d)	185.2 ± 8.1	192.3 ± 5.3	0.13
	Performance parameters ^3^ of broilers during the whole experimental period (day 0–35)			
	BW start (g) on day 0	46.9 ± 1.0	47.1 ± 0.6	0.70
	BW end (g) on day 35	2509.5 ± 75.9	2606.3± 63.2	0.05
	dBWG (g/d)	70.4 ± 2.2	73.1 ± 1.8	0.05
	FCR (g/g)	1.47 ± 0.02	1.45 ± 0.02	0.23
	dFI (g/d)	103.2 ± 4.0	106.1 ± 3.4	0.25
	FCRcorrected ^4^ (g/g)	1.39 ± 0.02	1.42 ± 0.02	**0.02**
	Area under the curve (AUC) of haptoglobin and interleukin-13 (IL-13) ^5^ during grower phase			
Immune response	Haptoglobin AUC ((ng/mL) × days)	13.1 ± 2.8	10.4 ± 2.5	**0.02**
	IL-13 AUC ((pg/mL) × days) ^6^	8.4 ± 2.3	5.1 ± 1.9	**<0.01**
	Haptoglobin and IL-13 concentrations ^7^ on day 14			
	Haptoglobin (ng/mL)	2.3 ± 0.8	1.8 ± 0.6	0.09 ^8^
	IL-13 (pg/mL)	61.8 ± 63.7	8.5 ± 5.2	<0.01 ^8^
	Haptoglobin and IL-13 concentrations on day 21			
	Haptoglobin (ng/mL)	1.4 ± 0.3	1.2 ± 0.3	0.06
	IL-13 (pg/mL)	18.2 ± 16.8	18.6 ± 19.0	0.41 ^8^
	Jejunal morphology ^7^ on day 14			
Intestinal morphology	Villi length (μm)	679.0 ± 70.5	642.4 ± 97.5	0.32
	Crypt depth (μm)	174.7 ± 17.7	186.3 ± 28.4	0.27
	V:C ^9^ ratio	4.0 ± 0.4	3.6 ± 0.7	0.15
	Villi size area (μm^2^)	95,878 ± 14,203	88,178 ± 13,977	0.21
	Goblet cell surface area (% of villi)	2.0 ± 0.6	2.1 ± 0.8	0.85
	Goblet cells per villi	197.3 ± 78.1	178.3 ± 66.5	0.54
	Goblet cell size (μm^2^)	10.6 ± 2.6	10.1 ± 1.1	0.62

^1^ Diet containing 0.8% CV dried powdered biomass. ^2^ *p*-value < 0.05 were considered significant and highlighted in bold. ^3^ Data are presented as mean with standard deviation (*n* = 6 pens per group). ^4^ FCR_corrected_ = FCR_0–35_ − ((BW_35_ − 2250)/50 × 100)). ^5^ Data are presented as mean with standard deviation (*n* = 12 birds per group). ^6^ Based on logarithmic transformation of IL-13 figures to normalize distribution. ^7^ Data are presented as mean with standard deviation (*n* = 12 birds per group; samples were taken from two birds per pen; each pen is an experimental unit). ^8^ Original values are presented here but logarithmic values were used for test as distributions were not normal. ^9^ Villi length to crypt depth.

## Data Availability

The data presented in this study are available on request from the corresponding author.

## Supplementary Materials

The following supporting information can be downloaded at: https://www.mdpi.com/article/10.3390/ani12091114/s1, Table S1: Nutrient composition of the *Chlorella vulgaris* biomass ingredient for 100 grams; Table S2: Control (CON) and *Chlorella vulgaris* (CV) diet formulations of the starter, grower, and finisher phase of the trial; Table S3: Control (CON) and *Chlorella vulgaris* (CV) diet calculated nutrient compositions of the starter, grower, and finisher phase of the trial.
