# Supplementary material for: Effect of Feeding 0.8% Dried Powdered Chlorella vulgaris Biomass on Growth Performance, Immune Response, and Intestinal Morphology during Grower Phase in Broiler Chickens
Source: Animals (Basel). 2022 Apr 26;12(9):1114. doi: 10.3390/ani12091114 (PMC9102271; doi:10.3390/ani12091114)
Supplement: Supplementary file 1 [file animals-12-01114-s001.zip › animals-1634340-supplementary.pdf]

animals

Communication

Effect of Feeding 0.8% Dried Powdered *Chlorella vulgaris* Biomass on Growth Performance, Immune Response, and Intestinal Morphology during Grower Phase in Broiler Chickens

Simon Roques, Sietse-Jan Koopmans, Annemarie Mens, Jan van Harn, Marinus van Krimpen † and Soumya Kanti Kar *

Wageningen Livestock Research, Wageningen University and Research, Wageningen 6708WD, the Netherlands.; simon.roques@wur.nl (S.R.); sietsejan.koopmans@wur.nl (S.-J.K.); annemarie.mens@wur.nl (A.M.); jan.vanharn@wur.nl (J.v.H.)
* Correspondence: soumya.kar@wur.nl
† Deceased 31st of December 2019.

Citation: Roques, S.; Koopmans, S.-J.; Mens, A.; van Harn, J.; van Krimpen, M.; Kar, S.K. Effect of Feeding 0.8% Dried Powdered *Chlorella vulgaris* Biomass on Growth Performance, Immune Response, and Intestinal Morphology during Grower Phase in Broiler Chickens. *Animals* **2022**, *12*, 1114. https://doi.org/10.3390/ani12091114

Academic Editor: Velmurugu Ravindran

Received: 25 February 2022
Accepted: 22 April 2022
Published: 26 April 2022

Publisher's Note: MDPI stays neutral with regard to jurisdictional claims in published maps and institutional affiliations.

Copyright: © 2022 by the authors. Licensee MDPI, Basel, Switzerland. This article is an open access article distributed under the terms and conditions of the Creative Commons Attribution (CC BY) license (https://creativecommons.org/licenses/by/4.0/).

Table S1. Nutrient composition of the *Chlorella vulgaris* biomass ingredient for 100 grams.

**Dry matter**	**g**	**95.5**
Energy	kCal	434
Crude Fats	g	14.2
Saturated fatty acids	g	6.7
Monounsaturated fatty acids	g	4.1
Polyunsaturated fatty acids	g	3.4
Carbohydrates	g	38.7
of which sugars	g	5.0
Fibres	g	32.9
Crude protein	g	22.7
Vitamins		
A (Retinol)	ug	180.0
D	ug	1.9
E (Tocopherol)	mg	13.9
C (Ascorbic Acid)	mg	83.8
B1 (Thiamine)	mg	0.3
B2 (Ribovlavine)	mg	0.6
B3 (Niacone)	mg	16.7
B6 (Pyridoxine)	mg	1.0
B11 (Folate)	ug	2.0
B5 (Pantothenic Acid)	mg	0.7
Minerals		
Potassium	mg	990.0
Calcium	mg	49.0
Phosphorus	mg	925.0
Magnesium	mg	87.0
Iron	mg	9.4
Manganese	mg	2.6
Zinc	mg	3.5
Fluorine	mg	< 0.5
Nitrite	mg	< 0.2
Sodium	mg	149.0

Table S2. Control (CON) and *Chlorella vulgaris* (CV) diet formulations of the starter, grower, and finisher phase of the trial.

Ingredients (%)	Starter		Grower		Finisher	
	CON	CV	CON	CV	CON	CV
*Chlorella vulgaris*_v2		0.80		0.80		0.80
Maize/Corn	35.00	35.00	35.00	35.00	32.50	32.50
Wheat	30.18	29.73	34.62	34.17	45.54	45.09
Soybean meal Hypro	23.92	23.64	19.08	18.80	11.92	11.64
Potato protein	2.59	2.57	4.01	3.99	4.97	4.95
Palm oil	1.24	1.22	2.50	2.50	0.31	0.29
Soy oil	2.00	2.00	0.50	0.48	1.00	1.00
Limestone (fine)	1.61	1.62	1.35	1.36	1.09	1.10
Natrium-Bicarbonate	0.42	0.40	0.37	0.35	0.39	0.37
Salt	0.05	0.03	0.05	0.03	0.04	0.03
Monocalcium phosphate	1.12	1.10	0.79	0.77	0.54	0.51
Potassium carbonate	0.04	0.04	0.04	0.04	0.27	0.27
Premix broiler [1]	0.50	0.50	0.50	0.50	0.50	0.50
L-Lysine HCL	0.33	0.33	0.26	0.27	0.27	0.27
DL-Methionine	0.29	0.29	0.24	0.25	0.20	0.20
L-Threonine	0.11	0.11	0.06	0.06	0.04	0.04
L-Isoleucine	0.01	0.02	0.03	0.03	0.03	0.03
L-Arginine	0.09	0.10	0.08	0.09	0.12	0.13
Phytase enzyme	0.50	0.50	0.50	0.50	0.26	0.26
Rovabio Excel AP (NSP-enzyme)	0.01	0.01	0.01	0.01	0.01	0.01
Total	100	100	100	100	100	100

[1] Composition of Premix: 12,000 IU vitamin A, 2400 IU vitamin D3, 50 IU vitamin E, 1.5 mg vitamin K3, 2 mg vitamin B1, 7.5 mg vitamin B2, 35 mg niacin amide, 12 mg d-pantothenic acid, 3.5 mg vitamin B6, 25 µg vitamin B12, 200 µg biotin, 460 mg choline chloride, 1 mg folic acid, 80 mg Fe (as $FeSO_4 \bullet 7H_2O$), 85 mg Mn (as MnO), 12 mg Cu (as $CuSO_4 \bullet 5H_2O$), 60 mg Zn (as $ZnSO_4 \bullet H_2O$), 0.8 mg I (as KI), 0.15 mg Se (as $Na_2SeO_3 \bullet 5H_2O$) and 125 mg anti-oxidant.

Table S3. Control (CON) and *Chlorella vulgaris* (CV) diet calculated nutrient compositions of the starter, grower, and finisher phase of the trial.

**Nutrient** [1]		**Starter**		**Grower**		**Finisher**	
		**CON**	**CV**	**CON**	**CV**	**CON**	**CV**
Dry matter	g	881	882	880	881	877	878
Crude ash	g	58	57	49	49	43	43
Crude protein	g	203	203	195	195	180	180
Crude Fat (Acid Hydrolysis)	g	61	62	59	60	41	42
Crude fibre	g	24	27	24	26	23	25
Starch	g	398	395	424	421	472	469
Calcium	g	9.20	9.20	7.60	7.60	5.90	5.90
Phosphate (total)	g	5.99	5.99	5.09	5.08	4.32	4.31
Retainable P	g	4.40	4.40	3.70	3.70	2.80	2.80
Potassium	g	8.13	8.13	7.26	7.26	7.26	7.26
Sodium	g	1.40	1.40	1.30	1.30	1.30	1.30
Chloride	g	1.55	1.55	1.50	1.50	1.50	1.50
Electrolyte balance	kg	225	225	200	200	200	200
Phytase	FTU [2]	500	500	500	500	260	260
Metabolizable Energy $_{broiler}$	MJ [3]	11.90	11.90	12.10	12.10	12.10	12.10
Dig. Lysine	g	11.31	11.31	10.50	10.50	9.51	9.51
Dig. methionine	g	5.69	5.72	5.27	5.29	4.72	4.74
Dig. Met+Cys	g	8.37	8.37	7.87	7.87	7.20	7.20
Dig. threonine	g	7.38	7.38	6.80	6.80	6.15	6.15
Dig. tryptophan	g	2.04	2.04	1.97	1.97	1.79	1.79
Dig. isoleucine	g	7.47	7.47	7.39	7.39	6.72	6.72
Dig. arginine	g	11.88	11.88	11.02	11.02	9.99	9.99
Dig. leucine	g	14.45	14.41	14.25	14.22	13.17	13.13
Dig. valine	g	8.16	8.16	8.07	8.07	7.49	7.49

[1] Diets were formulated using data on ingredient nutrient composition and nutrient digestibility coefficients according to the Central Bureau for Livestock Feeding (CVB, Lelystad, the Netherlands). [2] Phytase unit activity. [3] Megajoules
